# Supplementary material for: Neural signatures of emotional biases predict clinical outcomes in difficult-to-treat depression
Source: Res Dir Depress. 2024 Oct 1;1:e21. doi: 10.1017/dep.2024.6 (PMC11869767; doi:10.1017/dep.2024.6)
Supplement: Fennema et al. supplementary material [file S2976900024000069sup001.docx]

**Supplementary Online Content**

**Neural signatures of emotional biases predict clinical outcomes in difficult-to-treat depression**

Diede Fennema^1^, Gareth J. Barker^2^, Owen O’Daly^2^, Beata R. Godlewska^3,4^, Ewan Carr^5^, Kimberley Goldsmith^5^, Allan H. Young^1,6^, Jorge Moll^7^ & Roland Zahn^1,6,7*^

*^1^ Centre of Affective Disorders, Institute of Psychiatry, Psychology & Neuroscience, King’s College London, London, UK*

*^2^ Department of Neuroimaging, Institute of Psychiatry, Psychology & Neuroscience, King’s College London, London, UK*

*^3^ Psychopharmacology Research Unit, University Department of Psychiatry, University of Oxford, Oxford, UK*

*^4^ Oxford Health NHS Foundation Trust, Warneford Hospital, Oxford, UK*

*^5^ Department of Biostatistics and Health Informatics, Institute of Psychiatry, Psychology & Neuroscience, King’s College London, London, UK*

*^6^ National Service for Affective Disorders, South London and Maudsley NHS Foundation Trust, London, UK*

*^7^ Cognitive and Behavioral Neuroscience Unit, D’Or Institute for Research and Education (IDOR), Pioneer Science Program, Rio de Janeiro, Brazil*

* Corresponding author

Professor Roland Zahn (see address above)

E-mail: roland.zahn@kcl.ac.uk

Phone: 0044-(0)20 7848 0348

Fax: 0044-(0)20 7848 0298

Keywords: *depression; prediction; fMRI; prognosis; difficult-to-treat*

**Parts of this Supplementary Online Content have been adapted from a previously published one in Neuroimage:Clinical (doi: 10.1016/j.nicl.2023.103453).**

**Supplementary Methods**

*Additional inclusion/exclusion criteria*

In addition to the criteria mentioned in the main manuscript, participants were included if they met any of the following: aged 18 years and over, currently experiencing a major depressive episode (MDE) and at least moderately severe depressive syndrome on the Patient Health Questionnaire (PHQ-9; score ≥ 15) (Spitzer et al. 1999), and non-responders to at least two serotonergic antidepressants from the following list in current or previous episodes: citalopram, fluoxetine, sertraline, escitalopram, paroxetine, venlafaxine or duloxetine.

Participants were excluded if they met any of the following: previous prescription of mirtazapine or vortioxetine at therapeutic dose, MRI contraindications, currently receiving specialist psychiatric treatment, high suicide risk on the Mini International Neuropsychiatric Interview (MINI) suicidality screen (Sheehan et al. 1998), past diagnosis of schizophrenia or schizo-affective disorder, psychotic symptoms using clinical screening questions, bipolar disorder, at risk of being violent, drug or alcohol abuse over the last six months, suspected neurological condition, pregnancy or insufficient contraception in women of childbearing age and breastfeeding or within six months of giving birth.

*Recruitment and clinical assessment*

We recruited participants from September 2018 to March 2020 partly through a cluster-randomised feasibility clinical trial, the Antidepressant Advisor Study (ADeSS; NCT03628027), which evaluated the feasibility of a novel computerised decision support algorithm for antidepressant medications in patients with major depressive disorder (MDD) in primary care (Harrison et al. 2020; Harrison et al. 2022). Participants enrolled in the ADeSS trial were assigned to either i) use of a computerised decision-support tool by their general practitioner (GP) to assist with antidepressant choices, or ii) treatment-as-usual, and were asked to attend an optional MRI session. The computerised decision-support tool implemented National Institute for Health and Care Excellence guidelines, prompting GPs to increase the dose or switch to another antidepressant, and resembled standard care. Recruitment was halted due to the COVID-19 pandemic and recommenced in October 2020, using online advertising only, and was completed in August 2021.

As described in the trial protocol (Harrison et al. 2020), GP practices screened for patients with a history of treatment-resistance to antidepressant medications within their practice, i.e. non-responders to at least two serotonergic antidepressants in the current or previous episodes. Potential participants were approached for consent and, if given, asked to fill in a pre-screening questionnaire. Potentially eligible participants were invited for an in-depth assessment by the study team, which included a clinical assessment using the Structured Clinical Interview for DSM-5 (SCID) to establish a current MDD (First et al. 2015), a history of participants’ depressive episodes, their current and past antidepressant medications, and completing various clinical, behavioural and experimental measures.

A follow-up assessment was conducted to establish whether any changes in baseline measures had occurred. This visit took place around 14-18 weeks after enrolling in the study, which should allow observation of any treatment effect if there is one. The assessment included questions related to medications taken in the study period as well as various clinical and behavioural measures. The main clinical measures collected at baseline and follow-up were the Quick Inventory of Depressive Symptomology (16 items, self-rated; QIDS-SR16) (Rush et al. 2003), Maudsley Modified Patient Health Questionnaire (9 items; MM-PHQ-9) (Harrison et al. 2021), Generalised Anxiety Disorder (7 items; GAD-7) (Spitzer et al. 2006), Montgomery-Åsberg Depression Rating Scale (MADRS) (Montgomery and Asberg 1979), and Social and Occupational Functioning Assessment Scale (SOFAS, part of SCID) (First et al. 2015). Please refer to the ADeSS trial protocol for more details regarding these procedures (Fennema 2022; Harrison et al. 2020).

As the ADeSS trial was stopped due to the COVID-19 pandemic, an alternative recruitment route was employed to continue recruitment for the observational fMRI study. Trial adverts were posted online, with further dissemination of study adverts via university and institutional recruitment circulars. Interested participants were asked to complete a similar pre-screening questionnaire as those approached for the ADeSS trial. If potentially eligible, participants were invited for an in-depth assessment to confirm their eligibility. This group resembled the treatment-as-usual arm in the ADeSS trial, receiving standard care. For more details, please see Fennema (2022).

A total of 1,755 participants with a history of MDD showed interest in participating and completed a pre-screening questionnaire. Potentially eligible MDD participants (n = 89) for the ADeSS trial and the fMRI study were invited to attend an in-depth assessment. Of those, 45 participants enrolled in the fMRI study, attended their MRI session and completed the study. Of those 45 participants, ten participants were also part of the ADeSS trial (support tool arm: n = 4; treatment-as-usual arm: n = 6).

*Sample size*

As there was no previous comparable study from which effect sizes could be drawn, we carried out a sensitivity analysis showing that for determining an at least 20% above chance level prediction model performance, a minimum of n = 44 MDD patients was required to achieve 85% power at *p* = .05 using a binomial test.

*fMRI paradigms*

Participants were asked to complete three fMRI paradigms: the moral sentiment task, the subliminal faces task and a resting-state scan:

1. *Moral sentiment task, as described in Fennema et al. (2023)*

Participants were shown an optimised and shortened version of the fMRI paradigm outlined by Green et al. (2012) and Lythe et al. (2015). For details on the optimisation, please see Duan et al. (2023) and Fennema (2022). In brief, participants were shown 54 short written statements describing actions counter to social and moral values described by social concepts (e.g. impatient, dishonest) in which the agent was either the participant (self-agency condition [number of stimuli = 27]) or their best friend (other-agency condition [number of stimuli = 27]). Participants were asked to name their best friend prior to the scanning session to allow personalisation of the statements. Self- and other-agency conditions used the same social concepts (e.g. self-agency “Tom is dishonest towards Pete” and e.g. other-agency “Pete is dishonest towards Tom”). In addition, there were 27 low-level null events as a baseline condition, i.e. fixation of a visual pattern with no button press or other response required.

Stimuli were presented in an event-related design for a maximum of 5 seconds, within which time participants had to decide whether they would feel that the imagined behaviours were “quite unpleasant” or “mildly unpleasant” from their own perspective. The stimuli were presented in a pseudo-random order, presented at jittered intervals with a mean of 4000ms (with steps of 500ms). The total task time was 12 minutes and 9 seconds.

1. *Subliminal faces task, as described in Fennema, Barker, O'Daly, Duan, Godlewska, et al. (2024)*

Participants completed a backward masking task based on the fMRI paradigm outlined by Godlewska et al. (2018). Participants were shown pairs of faces, with a first “target” face (expressing a sad, happy, or neutral emotion), displayed for 34 milliseconds, and then immediately “masked” by a face of neutral expression, displayed for 66 milliseconds. The task followed a block design, with each participant being shown four blocks with sad faces, four blocks with happy faces and nine blocks with neutral faces. Each block cycled through ten target-mask pairs of faces, with the order varying for each block. The neutral (N) blocks were interleaved with sad (S) and happy (H) blocks, in one of two orders: N-S-N-H-N-S-H-N or N-H-N-S-N-H-N-S-N. The order of blocks was determined by pseudo-randomisation, with an even split within the MDD and control groups and across the total sample. After each block, there was a 10-second block of baseline fixation. The total task time was 8 minutes and 47 seconds.

1. *Resting-state scan, as described in Fennema, Barker, O'Daly, Duan, Carr, et al. (2024)*

The resting-state scan was based on the methodology as outlined by Dunlop et al. (2017). Participants were shown a fixation cross on the screen and were instructed to keep their eyes open and let their mind wander while focusing on the cross. The total scan time was 7 minutes and 24 seconds.

*Image acquisition*

Image acquisition was carried out on an MR750 3T MR system (GE Healthcare, Chicago, USA), using a Nova Medical 32-channel head coil. The scanning session started with approximately 20 minutes of structural imaging, acquiring T1-weighted, T2*-weighted and Fluid-Attenuated Inversion Recovery (FLAIR) images, followed by approximately 30 minutes of fMRI paradigms (moral sentiment, subliminal faces and resting-state). While in the MRI scanner, the participant’s head motion was restricted using padding, and heart rate and respiration rate measurements were recorded via a manufacturer-supplied finger pulse sensor (peripheral plethysmograph) and respiratory belt, respectively. A mirror fitted to the head coil allowed participants to view visual stimuli presented during image acquisition, as stimuli were projected onto a screen located behind the participant’s head. Verbal instructions were communicated via the MRI intercom, using a pre-defined script to ensure consistency between participants.

High-resolution anatomical images were acquired with a 3D Inversion Recovery prepared Spoiled Gradient Echo sequence (IR-SPGR; repetition time (TR) = 7.3 ms; echo time (TE) = 3.02 ms; inversion time (TI) = 400 ms; matrix = 256 x 256; excitation flip angle = 11 degrees; field-of-view (FOV) = 270 mm; slice thickness = 1.2 mm, 196 slices). Images for incidental findings review were acquired using a 2D Fast-Recovery Fast Spin-Echo (TR = 4380 ms; TE = 64.85 ms; matrix = 320 x 256; refocusing flip angle = 111 degrees; FOV = 240; 2 mm contiguous slices, 72 slices) and 2D FLAIR sequence (TR = 8000 ms; TE = 128.41 ms; matrix = 256 x 128; refocusing flip angle = 111 degrees; FOV = 220; 4 mm continuous slices, 36 slices) and checked by a neuroradiologist at King’s College London Hospital for any significant brain abnormalities that might warrant follow up, independent of additional, internal checks by the study team.

For all three paradigms, shimming was automatically applied as part of the scanner’s “pre-scan” procedures, and four additional volumes were acquired and automatically discarded at the start of each fMRI run, allowing for T1 equilibration effects:

1. *Moral sentiment task, as described in Fennema et al. (2023)*

Functional image acquisition was obtained in the anterior commissure – posterior commissure plane, with slices running top to bottom, using a T2*-weighted echo-planar imaging blood-level oxygen-dependent (BOLD) sequence (TR = 2000ms; TE = 20ms; matrix = 64x64; FOV = 211mm; flip angle = 75 degrees; slice thickness = 2.9mm, slice gap = 0.1mm, inter-slice distance = 3mm, 41 slices, 368 volumes).

1. *Subliminal faces task, as described in Fennema, Barker, O'Daly, Duan, Godlewska, et al. (2024)*

Functional image acquisition was obtained parallel to the anterior commissure – posterior commissure plane, with slices running top to bottom, using a standard T2*-weighted echo-planar imaging BOLD sequence (TR = 2000 ms; TE = 30 ms; matrix = 64 x 64; FOV = 240 mm; flip angle = 75 degrees; slice thickness = 3 mm, slice gap = 0.3 mm, inter-slice distance = 3.3 mm, 41 slices, 267 volumes).

1. *Resting-state scan, as described in Fennema, Barker, O'Daly, Duan, Carr, et al. (2024)*

Resting-state echo-planar images were acquired using a sequence which was optimised for the detection of ventral frontal signal (222 volumes; 41 slices; descending sequential acquisition; TR = 2000ms; TE = 20ms; matrix = 64 x 64; FOV = 211mm; flip angle = 75 degrees; slice thickness = 2.9mm, slice gap = 0.1mm, inter-slice distance = 3mm).

*Image analysis*

Statistical Parametric Mapping (SPM) 12 (http://www.fil.ion.ucl.ac.uk/spm12) was used for BOLD effect analysis and psychophysiological interaction (PPI) analysis (moral sentiment paradigm, subliminal faces paradigm), while Data Processing Assistant for Resting-State fMRI (DPARSF) (Chao-Gan and Yu-Feng 2010) was used for resting-state analysis.

1. *Moral sentiment task, as described in Fennema et al. (2023)*

Standard pre-processing steps were followed: functional images were realigned, unwarped and co-registered to the participant’s T1 images. These images were normalised to the co-registered T1 image and resliced at a voxel size of 3 x 3 x 3 mm. A smoothing kernel of full-width half-maximum equal to 6 mm was used. No slice timing correction was applied. Motion correction was applied in the form of censoring, i.e. identifying outliers based on framewise displacement and regressing them from the fMRI timeseries. Framewise displacement was calculated using Brain and Mind Lab (BRAMILA) tools (https://github.com/spunt/bspm/blob/master/ thirdparty/bramila/bramila_framewiseDisplacement.m) to identify outliers regarding motion. Any framewise displacement of $\geq$0.5 mm was marked as a spike in movement and scan nulling regressors were added to the standard six motion parameters describing movement by rotation and translation to account for the spike(s). Participants with spikes in more than 25% of the functional images overall were deemed to have moved too much and were excluded from the analysis. We chose a threshold of 25% of motion-contaminated volumes in combination with the threshold of any framewise displacement of $\geq$0.5 mm as a trade-off between retaining patient data with reasonable quality and avoiding overfitting with too many scanning nulling regressors.

At the individual level, BOLD effects were modelled for the self-agency condition, other-agency condition and null event, with an event duration of 0 seconds. Movement parameters (i.e. six parameters describing movement by rotation and translation in three dimensions each, plus any scan nulling regressors) were included as covariates. No time and dispersion derivatives were modelled.

Connectivity was determined using PPI analysis. We extracted the signal from our pre-registered seed region, i.e. the right superior anterior temporal lobe (RSATL; Montreal Neurological Institute [MNI] coordinates: x = 58, y = 0, z = -12; 6 mm sphere), and created interaction terms for the psychological variable (main effect of condition, i.e. self-agency vs. fixation and other-agency vs. fixation) with the physiological variable (the right superior anterior temporal lobe signal time course irrespective of condition).

1. *Subliminal faces task, as described in Fennema, Barker, O'Daly, Duan, Godlewska, et al. (2024)*

Standard pre-processing steps were followed: functional images were realigned, unwarped and co-registered to the participant’s T1 images. These images were normalised to the co-registered T1 image and resliced at a voxel size of 3 x 3 x 3 mm. A smoothing kernel of full-width half-maximum equal to 6 mm was used. No slice timing correction was applied. Motion correction was applied in the form of censoring. Framewise displacement was calculated using BRAMILA tools to identify outliers regarding motion. Any framewise displacement of $\geq$1 mm was marked as a spike in movement and scan nulling regressors were added to the standard six motion parameters describing movement by rotation and translation to account for the spike(s). Participants with spikes in more than 10% of the functional images overall were deemed to have moved too much and were excluded from the analysis. We chose a threshold of 10% of motion-contaminated volumes in combination with the threshold of any framewise displacement of $\geq1$ mm as a trade-off between retaining patient data with reasonable quality and avoiding overfitting with too many scanning nulling regressors.

Additional noise correction was applied: the MATLAB PhysIO toolbox was used to partially mitigate the impact of physiological noise (Kasper et al. 2017) (version R2021a-v8.0.0, open-source code available as part of the Translational Algorithms for Psychiatry-Advancing Science software collection (Frassle et al. 2021): https://www.translationalneuromodeling .org/tapas). Heart rate and respiration rate measurements were used in a retrospective image correction (RETROICOR) model, using Fourier expansions of different orders for the estimated phases of cardiac pulsation (third order), respiration (fourth order) and cardio-respiratory interactions (first order) (Harvey et al. 2008).

BOLD effects were modelled for each of the emotion blocks, i.e. sad, happy and neutral. Baseline fixation was not modelled to avoid overspecification of the model. Nuisance regressors created by the PhysIO toolbox, i.e. physiological noise regressors and motion-related regressors, were included as covariates. Contrasts were created to examine the subtraction-based difference between sad and happy faces (sad vs. happy).

1. *Resting-state scan, as described in Fennema, Barker, O'Daly, Duan, Carr, et al. (2024)*

The resting-state fMRI pre-processing followed a similar approach to that outlined in Workman et al. (2016), using DPARSF and Artifact Detection Tools (ART). SPM8 was used for pre-processing steps to ensure compatibility with DPARSF.

Functional resting-state echo-planar images (EPIs) and IR-SPGR anatomical images underwent standard pre-processing steps in DPARSF. ART was used to flag spikes in motion, i.e. framewise signal intensity > 3 standard deviation from the global mean and framewise head displacement > 1mm, and to create nulling regressors. Participants with spikes in more than 10% of the functional images were deemed to have moved too much and were excluded from the analysis.

In addition, the MATLAB PhysIO toolbox was used to partially mitigate the impact of physiological noise (Kasper et al. 2017). Heart rate and respiration rate measurements were used in a RETROICOR model, using Fourier expansions of different orders for the estimated phases of cardiac pulsation (second order), respiration (second order) and cardio-respiratory interactions (first order) (Glover et al. 2000).

Following this initial pre-processing, EPIs underwent linear detrending and nuisance covariates regression (6 motion parameters (Bright and Murphy 2015), white matter signal, cerebrospinal fluid signal, ART regressors and PhysIO regressors) and normalisation using non-linear transformation parameters derived during segmentation. Band-pass filtering was applied to retain frequencies between 0.01 and 0.08 Hz.

Functional connectivity maps were computed using the fully pre-processed functional images for each participant by correlating the average time course within the seed region (i.e. subgenual frontal cortex [Brodmann Area [BA] 25]) with the time course of each voxel within the brain, which were Fisher Z-transformed.

*Description of treatment change*

As part of the study, participants were encouraged to book an appointment with their GP to review their treatment. Exploratory analysis was undertaken to attribute a reduction in depressive symptoms to a change in pharmacological treatment, i.e. an increase in dose or a change to another medication. More specifically, participants were classified as follows: i) no change, i.e. participants who did not make any changes to their treatment, stopped taking their antidepressant, or lowered the dose of their current antidepressant; ii) minimal change, i.e. participants who increased their current antidepressant from an effective dose to a higher dose, or who changed to another antidepressant at an ineffective dose; and iii) relevant change, i.e. participants who increased their current antidepressant from an ineffective dose to an effective dose, or who changed to another antidepressant at an effective dose.

Some participants had more than one change in their treatment during the follow-up period. In those cases, the change most relevant for the clinical outcome measure was used, which was usually the change occurring closest to the follow-up assessment. Moreover, the change to another antidepressant at an effective dose or an increase in dose had to have lasted at least two weeks prior to the follow-up assessment to be counted a treatment trial, which is in line with the Maudsley Staging Method (Fekadu et al. 2018).

*Exploratory prediction models*

In addition to our main multivariate prediction model, we ran exploratory models to assess the contribution of the pre-registered fMRI measures individually, with baseline MM-PHQ-9 as a covariate: “self-blaming biases” with a focus on self-blame-selective connectivity between the right superior anterior temporal lobe and the posterior subgenual cortex (BA25), “negative perceptual biases” with a focus on bilateral amygdala BOLD activation for subliminal sad vs happy faces, and “subgenual resting-state networks” with a focus on subgenual resting-state connectivity with the ventrolateral prefrontal cortex/insula (BA47).

Moreover, we ran an exploratory “pre-registration” model which included three additional neural measures as originally outlined in our pre-registered protocol (NCT04342299), i.e. functional resting-state subgenual cortex connectivity with the left ventromedial prefrontal cortex (BA10) and with the dorsal midbrain, as well as pregenual anterior cingulate cortex BOLD activation for subliminal sad vs happy faces.

**Supplementary Results**

*Exploratory prediction models*

There was no association between baseline MM-PHQ-9 and the pre-registered fMRI measures, i.e. self-blame-selective connectivity between the right superior anterior lobe and the posterior subgenual cortex (BA25; *r*_s_ [30] = -.19, *p* = .32), bilateral amygdala BOLD activation to sad vs happy (*r*_s_ [30] = .03, *p* = .86) and resting-state connectivity between the subgenual cortex and ventrolateral prefrontal cortex/insula (*r*_s_ [30] = -.18, *p* = .33).

The “self-blaming biases” model explained 12% of the variance in QIDS-SR16 percentage (*F*[2,35] = 2.45, *p* = .10, *R^2^* = .12, *R^2^*_adjusted_ = .07; Supplementary Table 3). Self-blame-selective connectivity between the right superior anterior temporal lobe and the posterior subgenual cortex (BA25) negatively contributed to the variance in QIDS-SR16 percentage change (partial *β* = -9.59, *t*[35] = -2.10), while there was a positive contribution by baseline MM-PHQ-9 (partial *β* = 3.17, *t*[35] = .42).

The “negative perceptual biases” model explained 18% of the variance in QIDS-SR16 percentage (*F*[2,35] = 3.79, *p* = .03, *R^2^* = .18, *R^2^*_adjusted_ = .13; Supplementary Table 3). Bilateral amygdala BOLD activation to sad vs happy subliminal faces positively contributed to the variance in QIDS-SR16 percentage change (partial *β* = 13.87, *t*[35] = 2.66), as did baseline MM-PHQ-9 (partial *β* = 7.19, *t*[35] = .98).

The “subgenual resting-state networks” model explained 12% of the variance in QIDS-SR16 percentage (*F*[2,35] = 2.28, *p* = .12, *R^2^* = .12, *R^2^*_adjusted_ = .07; Supplementary Table 3). Resting-state connectivity between the subgenual cortex and ventrolateral prefrontal cortex/insula negatively contributed to the variance in QIDS-SR16 percentage change (partial *β* = -9.27, *t*[35] = -2.01), while there was a positive contribution by baseline MM-PHQ-9 (partial *β* = 4.21, *t*[35] = .56).

The “pre-registration” model explained 33% of the variance in QIDS-SR16 percentage (*F*[7,30] = 2.13, *p* = .07, *R^2^* = .33, *R^2^*_adjusted_ = .18; Supplementary Table 3). Baseline MM-PHQ-9 positively contributed to the variance in QIDS-SR16 (partial *β* = 3.20, *t*[30] = .43), while there was a negative contribution by self-blame-selective connectivity between the right superior anterior temporal lobe and the posterior subgenual cortex (BA25) (partial *β* = -7.53, *t*[30] = -1.65). Bilateral amygdala and pregenual anterior cingulate cortex BOLD to sad vs happy subliminal faces both positively contributed to the variance in QIDS-SR16 percentage (partial *β* = 11.06, *t*[30] = 1.99 and partial *β* = 2.62, *t*[30] = .49, respectively), as well as resting-state subgenual functional connectivity with the ventromedial prefrontal cortex (partial *β* = .64, *t*[30] = .17) and with the dorsal midbrain (partial *β* = 2.69, *t*[30] = .63). Resting-state subgenual functional connectivity with the ventrolateral prefrontal cortex/insula negatively contributed to the variance in QIDS-SR16 (partial *β* = -7.58, *t*[30] = -1.70).

*Exploratory findings responders vs. non-responders*

Of the predictor variables to explore response vs. non-response (Supplementary Table 4), increased functional connectivity between the bilateral subgenual cortex and left VLPFC/insula was significantly associated with an increased likelihood of response (OR [95% CI] = 2.87 [1.05, 7.84], *p =* .04). Increased self-blame-selective connectivity between the right superior anterior temporal lobe and the posterior subgenual cortex (BA25) (OR [95% CI] = 1.54 [.53, 4.45], *p* = .08) and higher baseline MM-PHQ-9 scores (OR [95% CI] = 6.36 [.65, 61.82]) were associated with an increased likelihood of response, whereas lower bilateral amygdala BOLD activation for sad vs happy subliminal faces was associated with a lower likelihood of response (OR [95% CI] = .226 [.04, 1.21]).

**Supplementary Tables**

**Supplementary Table 1.** Treatment during follow-up period (n=38).

| Characteristic | n (%) |
| --- | --- |
| Main change |  |
| No change in antidepressant | 22 (58%) |
| Stopped antidepressant | 5 (13%) |
| Lowered dose of antidepressant | 0 (0%) |
| Increase from effective dose to higher effective dose | 5 (13%) |
| Increase from ineffective dose to effective dose | 0 (0%) |
| Change to another antidepressant at effective dose | 4 (11%) |
| Change to another antidepressant at ineffective dose | 2 (5%) |
| Main antidepressant |  |
| SSRI | 31 (82%) |
| *Sertraline* | 8 (21%) |
| *Citalopram* | 5 (13%) |
| *Escitalopram* | 4 (11%) |
| *Fluoxetine* | 3 (8%) |
| *Venlafaxine (≤ 150mg)* | 6 (16%) |
| SNRI | 4 (11%) |
| *Duloxetine* | 2 (5%) |
| *Venlafaxine (> 150mg)* | 2 (5%) |
| Mirtazapine | 3 (8%) |
| Tricyclic antidepressant | 1 (3%) |
| Other antidepressant | 0 (0%) |
| Add-on treatment | 5 (13%) |
| Change in mental health service use |  |
| Started accessing mental health service | 8 (21%) |
| Continued care in mental health service | 8 (21%) |
| Stopped mental health treatment | 2 (5%) |
| Type of mental health service use |  |
| *CBT* | 3 (8%) |
| *Psychotherapy* | 5 (13%) |
| *Psychoanalysis* | 1 (3%) |
| *Counselling* | 2 (5%) |
| *Other* | 5 (13%) |
| Percentages may not add up to 100 due to rounding. MDD = major depressive disorder; SSRI = selective serotonin reuptake inhibitor; SNRI = selective noradrenaline reuptake inhibitor; CBT = cognitive behavioural therapy. | |

**Supplementary Table 2.** Descriptive statistics for clinical symptom measures at baseline and follow-up (n=38).

|  | Baseline  (mean ± SD; min – max) | Follow-up  (mean ± SD; min – max) | Difference [95% CI] | *t* value | *p* value |
| --- | --- | --- | --- | --- | --- |
| QIDS-SR16 | 17.3 ± 3.5; 10 – 23 | 13.2 ± 5.6; 4 – 24 | -4.1 [-5.8, -2.4] | -4.80 | <.001 |
| MM-PHQ-9 | 18.7 ± 4.7; 8 – 27 | 14.0 ± 7.8; 0 – 27 | -4.7 [-6.9, -2.5] | -4.28 | <.001 |
| GAD-7 ^a^ | 11.3 ± 4.3; 1 – 21 | 10.4 ± 5.7; 0 – 21 | -1.1 [-3.0, 0.8] | -1.21 | .12 |
| MADRS | 31.5 ± 4.9; 22 – 42 | 23.8 ± 10.9; 3 – 44 | -7.7 [-10.6, -4.7] | -5.30 | <.001 |
| SOFAS | 53.7 ± 5.4; 33 – 61 | 58.3 ± 11.2; 33 – 85 | 4.6 [1.7, 7.5] | 3.26 | .001 |
| ^a^ Missing follow-up data for one participant.  MDD = major depressive disorder; CI = confidence interval; QIDS-SR16 = Quick Inventory of Depressive Symptomatology – self-rated, 16 items; MM-PHQ-9 = Maudsley Modified Personal Health Questionnaire, 9 items; GAD-7 = Generalised Anxiety Disorder, 7 items; MADRS = Montgomery-Åsberg Depression Rating Scale; SOFAS = Social and Occupational Functioning Assessment Scale. M = mean; SD = standard deviation; min = minimum; max = maximum. | | | | | |

**Supplementary Table 3.** Exploratory prediction models of clinical outcomes in depression.

|  | Model parameters | | | | Overall model | |
| --- | --- | --- | --- | --- | --- | --- |
|  | ***β*** | **SE** | ***t*** | ***p*** | ***R*^2^** | ***p*** |
| Pre-registration (n=38) |  |  |  |  | .33 | .07 |
| Baseline MM-PHQ-9 | 3.20 | 7.40 | .43 | .67 |  |  |
| Self-blame-selective RSATL-BA25 connectivity | -7.53 | 4.56 | -1.65 | .11 |  |  |
| Bilateral amygdala BOLD activation for sad vs happy subliminal faces | 11.06 | 5.56 | 1.99 | .06 |  |  |
| Pregenual anterior cingulate cortex BOLD activation for sad vs happy subliminal faces | 2.62 | 5.31 | .49 | .63 |  |  |
| Resting-state subgenual cortex-VLPFC/insula functional connectivity | -7.58 | 4.45 | -1.70 | .10 |  |  |
| Resting-state subgenual cortex-VMPFC functional connectivity | .64 | 3.69 | .17 | .86 |  |  |
| Resting-state subgenual cortex-dorsal midbrain functional connectivity | 2.69 | 4.28 | .63 | .53 |  |  |
| Self-blaming biases (n=38) |  |  |  |  | .12 | .10 |
| Baseline MM-PHQ-9 | 3.17 | 7.60 | .42 | .68 |  |  |
| Self-blame-selective RSATL-BA25 connectivity | -9.59 | 4.57 | -2.10 | .04* |  |  |
| Negative perceptual biases (n=38) |  |  |  |  | .18 | .03* |
| Baseline MM-PHQ-9 | 7.19 | 7.32 | .98 | .33 |  |  |
| Bilateral amygdala BOLD activation for sad vs happy subliminal faces | 13.87 | 5.22 | 2.66 | .01* |  |  |
| Subgenual resting-state networks (n=38) |  |  |  |  | .12 | .12 |
| Baseline MM-PHQ-9 | 4.21 | 7.58 | .56 | .58 |  |  |
| Resting-state subgenual cortex-VLPFC/insula functional connectivity | -9.27 | 4.60 | -2.01 | .05 |  |  |
| High quality fMRI (n=30) |  |  |  |  | .43 | .01* |
| Baseline MM-PHQ-9 | 4.11 | 7.59 | .54 | .59 |  |  |
| Self-blame-selective RSATL-BA25 connectivity | -9.88 | 4.90 | -2.02 | .06 |  |  |
| Bilateral amygdala BOLD activation for sad vs happy subliminal faces | 9.56 | 6.25 | 1.53 | .14 |  |  |
| Resting-state subgenual cortex-VLPFC/insula functional connectivity | -11.86 | 4.68 | -2.54 | .02* |  |  |
| * significant at *p* < .05 threshold, two-tailed. SE = standard error; MM-PHQ-9 = Maudsley Modified Patient Health Questionnaire, 9 items; GAD-7 = Generalised Anxiety Disorder, 7 items; RSATL = right superior anterior temporal lobe; BA = Brodmann Area; BOLD = blood-oxygen level-dependent; VLPFC = ventrolateral prefrontal cortex; VMPFC = ventromedial prefrontal cortex. | | | | | | |

**Supplementary Table 4.** Logistic regression of response vs. non-response in depression (n=38).

| Predictor variable | *β* | SE | Wald | *p* | Odds Ratio  [95% CI] |
| --- | --- | --- | --- | --- | --- |
| Baseline MM-PHQ-9 | 1.85 | 1.16 | 2.54 | .11 | 6.36  [.65, 61.82] |
| Self-blame-selective RSATL-BA25 connectivity | .43 | .54 | .64 | .43 | 1.54  [.53, 4.45] |
| Bilateral amygdala BOLD activation for sad vs happy subliminal faces | -1.49 | .86 | 3.03 | .08 | .23  [.04, 1.21] |
| Resting-state subgenual cortex-VLPFC/insula functional connectivity | 1.05 | .51 | 4.21 | .04* | 2.87  [1.05, 7.84] |
| * significant at *p* < .05 threshold, two-tailed. SE = standard error; CI = confidence interval; MM-PHQ-9 = Maudsley Modified Patient Health Questionnaire, 9 items; GAD-7 = Generalised Anxiety Disorder, 7 items; RSATL = right superior anterior temporal lobe; BA = Brodmann Area; BOLD = blood-oxygen level-dependent; VLPFC = ventrolateral prefrontal cortex. | | | | | |

**References**

**Bright MG and Murphy K** (2015) Is fMRI "noise" really noise? Resting state nuisance regressors remove variance with network structure. *Neuroimage* **114,** 158-169. <https://doi.org/10.1016/j.neuroimage.2015.03.070>.

**Chao-Gan Y and Yu-Feng Z** (2010) DPARSF: A MATLAB Toolbox for "pipeline" data analysis of resting-state fMRI. *Front Syst Neurosci* **4,** 13. <https://doi.org/10.3389/fnsys.2010.00013>.

**Duan S, Valmaggia L, Fennema D, Moll J and Zahn R** (2023) Remote virtual reality assessment elucidates self-blame-related action tendencies in depression. *J Psychiatr Res* **161,** 77-83. <https://doi.org/10.1016/j.jpsychires.2023.02.031>.

**Dunlop BW, Rajendra JK, Craighead WE, Kelley ME, McGrath CL, Choi KS, Kinkead B, Nemeroff CB and Mayberg HS** (2017) Functional connectivity of the subcallosal cingulate cortex and differential outcomes to treatment with cognitive-behavioral therapy or antidepressant medication for major depressive disorder. *Am J Psychiatry* **174**(6)**,** 533-545. <https://doi.org/10.1176/appi.ajp.2016.16050518>.

**Fekadu A, Donocik JG and Cleare AJ** (2018) Standardisation framework for the Maudsley staging method for treatment resistance in depression. *BMC Psychiatry* **18**(1)**,** 100. <https://doi.org/10.1186/s12888-018-1679-x>.

**Fennema D** (2022) Neural signatures of emotional biases and prognosis in treatment-resistant depression. PhD, King's College London.

**Fennema D, Barker GJ, O'Daly O, Duan S, Carr E, Goldsmith K, Young AH, Moll J and Zahn R** (2023) Self-blame-selective hyper-connectivity between anterior temporal and subgenual cortices predicts prognosis in major depressive disorder. *NeuroImage: Clinical* **39,** 103453. <https://doi.org/10.1016/j.nicl.2023.103453>.

**Fennema D, Barker GJ, O'Daly O, Duan S, Carr E, Goldsmith K, Young AH, Moll J and Zahn R** (2024) The role of subgenual resting-state connectivity networks in predicting prognosis in major depressive disorder. *Biological Psychiatry: Global Open Science* **4**(3)**,** 100308. <https://doi.org/10.1016/j.bpsgos.2024.100308>.

**Fennema D, Barker GJ, O'Daly O, Duan S, Godlewska BR, Goldsmith K, Young AH, Moll J and Zahn R** (2024) Neural responses to facial emotions and subsequent clinical outcomes in difficult-to-treat depression. *Psychol Med*. <https://doi.org/10.1017/S0033291724001144>.

**First MB, Williams JBW, Karg RS and Spitzer RL** (2015) *Structured Clinical Interview for DSM-5 - Research Version (SCID-5 for DSM-5, Research Version; SCID-5-RV, Version 1.0.0)*. Arlington, VA: American Psychiatric Association.

**Frassle S, Aponte EA, Bollmann S, Brodersen KH, Do CT, Harrison OK, Harrison SJ, Heinzle J, Iglesias S, Kasper L, Lomakina EI, Mathys C, Muller-Schrader M, Pereira I, Petzschner FH, Raman S, Schobi D, Toussaint B, Weber LA, Yao Y and Stephan KE** (2021) TAPAS: An open-source software package for translational neuromodeling and computational psychiatry. *Front Psychiatry* **12,** 680811. <https://doi.org/10.3389/fpsyt.2021.680811>.

**Glover GH, Li TQ and Ress D** (2000) Image-based method for retrospective correction of physiological motion effects in fMRI: RETROICOR. *Magn Reson Med* **44**(1)**,** 162-167. <https://doi.org/10.1002/1522-2594(200007)44:1><162::aid-mrm23>3.0.co;2-e.

**Godlewska BR, Browning M, Norbury R, Igoumenou A, Cowen PJ and Harmer CJ** (2018) Predicting treatment response in depression: the role of anterior cingulate cortex. *Int J Neuropsychopharmacol* **21**(11)**,** 988-996. <https://doi.org/10.1093/ijnp/pyy069>.

**Green S, Lambon Ralph MA, Moll J, Deakin JF and Zahn R** (2012) Guilt-selective functional disconnection of anterior temporal and subgenual cortices in major depressive disorder. *Arch Gen Psychiatry* **69**(10)**,** 1014-1021. <https://doi.org/10.1001/archgenpsychiatry.2012.135>.

**Harrison P, Carr E, Goldsmith K, Young AH, Ashworth M, Fennema D, Barrett B and Zahn R** (2020) Study protocol for the antidepressant advisor (ADeSS): a decision support system for antidepressant treatment for depression in UK primary care: a feasibility study. *BMJ Open* **10**(5)**,** e035905. <https://doi.org/10.1136/bmjopen-2019-035905>.

**Harrison P, Carr E, Goldsmith K, Young AH, Ashworth M, Fennema D, Duan S, Barrett B and Zahn R** (2022) The Antidepressant Advisor (ADeSS): a decision support system for antidepressant treatment for depression in UK primary care - a feasibility study. In.

**Harrison P, Walton S, Fennema D, Duan S, Jaeckle T, Goldsmith K, Carr E, Ashworth M, Young AH and Zahn R** (2021) Development and validation of the Maudsley Modified Patient Health Questionnaire (MM-PHQ-9). *BJPsych Open* **7**(4)**,** e123. <https://doi.org/10.1192/bjo.2021.953>.

**Harvey AK, Pattinson KT, Brooks JC, Mayhew SD, Jenkinson M and Wise RG** (2008) Brainstem functional magnetic resonance imaging: disentangling signal from physiological noise. *J Magn Reson Imaging* **28**(6)**,** 1337-1344. <https://doi.org/10.1002/jmri.21623>.

**Kasper L, Bollmann S, Diaconescu AO, Hutton C, Heinzle J, Iglesias S, Hauser TU, Sebold M, Manjaly ZM, Pruessmann KP and Stephan KE** (2017) The PhysIO toolbox for modeling physiological noise in fMRI data. *J Neurosci Methods* **276,** 56-72. <https://doi.org/10.1016/j.jneumeth.2016.10.019>.

**Lythe KE, Moll J, Gethin JA, Workman CI, Green S, Lambon Ralph MA, Deakin JF and Zahn R** (2015) Self-blame-selective hyperconnectivity between anterior temporal and subgenual cortices and prediction of recurrent depressive episodes. *JAMA Psychiatry* **72**(11)**,** 1119-1126. <https://doi.org/10.1001/jamapsychiatry.2015.1813>.

**Montgomery SA and Asberg M** (1979) A new depression scale designed to be sensitive to change. *Br J Psychiatry* **134,** 382-389. <https://doi.org/10.1192/bjp.134.4.382>.

**Rush AJ, Trivedi MH, Ibrahim HM, Carmody TJ, Arnow B, Klein DN, Markowitz JC, Ninan PT, Kornstein S, Manber R, Thase ME, Kocsis JH and Keller MB** (2003) The 16-item Quick Inventory of Depressive Symptomatology (QIDS), clinician rating (QIDS-C), and self-report (QIDS-SR): a psychometric evaluation in patients with chronic major depression. *Biol Psychiatry* **54**(5)**,** 573-583. <https://doi.org/10.1016/s0006-3223(02)01866-8>.

**Sheehan DV, Lecrubier Y, Sheehan KH, Amorim P, Janavs J, Weiller E, Hergueta T, Baker R and Dunbar GC** (1998) The Mini-International Neuropsychiatric Interview (M.I.N.I.): the development and validation of a structured diagnostic psychiatric interview for DSM-IV and ICD-10. *J Clin Psychiatry* **59,** 22-33.

**Spitzer RL, Kroenke K, Williams JBW and PHQPCSG** (1999) Validation and utility of a self-report version of PRIME-MD: The PHQ primary care study. *JAMA* **282**(18)**,** 1737-1744.

**Spitzer RL, Kroenke K, Williams JBW and Lowe B** (2006) A brief measure for assessing generalised anxiety disorder: the GAD-7. *Arch Intern Med* **166**(10)**,** 1092-1097. <https://doi.org/10.1001/archinte.166.10.1092>.

**Workman CI, Lythe KE, McKie S, Moll J, Gethin JA, Deakin JF, Elliott R and Zahn R** (2016) Subgenual cingulate-amygdala functional disconnection and vulnerability to melancholic depression. *Neuropsychopharmacology* **41**(8)**,** 2082-2090. <https://doi.org/10.1038/npp.2016.8>.
